# Efficacy of a brief online mindfulness-based intervention on the psychological well-being of health care professionals and trainees during the COVID-19 pandemic: A mixed method design

**DOI:** 10.4102/hsag.v26i0.1682

**Published:** 2021-09-30

**Authors:** Iram Osman, Shaista Hamid, Veena S. Singaram

**Affiliations:** 1 School of Clinical Medicine, College of Health Sciences, University of KwaZulu-Natal, Durban, South Africa; 2 Phoenix Assessment and Therapy Center, KwaZulu-Natal Department of Health, Durban, South Africa

**Keywords:** mindfulness, stress, burnout, COVID-19, mental health, self-care, health professionals

## Abstract

**Background:**

During the coronavirus disease 2019 (COVID-19) pandemic, health professionals were pushed to the front line of a global health crisis unprepared and resource constrained, which affected their mental well-being.

**Aim:**

This study aimed to investigate the effectiveness of a brief online mindfulness-based intervention (MBI) on stress and burnout for health professionals training and working in South Africa during the COVID-19 crisis.

**Setting:**

The context of the study is the overburdened, under-resourced health care system in South Africa during a global pandemic.

**Methods:**

A mixed method framework was adopted for this study. The quantitative data was analysed using descriptive analysis and the participants’ qualitative experiences were interpreted using interpretative phenomenological analysis.

**Results:**

Forty-seven participants took part in this study. The study found a statistically significant (*p* < 0.05) reduction in stress levels and emotional exhaustion as well as an increase in mindful awareness and feelings of personal accomplishment after the intervention. The participants’ shared experiences were analysed in two parts. The pre-intervention analysis presented with central themes of loss of control and a sense of powerlessness because of COVID-19. The post-intervention analysis comprised themes of a sense of acquired control and empowerment through increased mindfulness.

**Conclusions:**

The study found that a brief online MBI can be associated with reduced levels of stress and burnout as well as an increased sense of control and empowerment, felt both personally and professionally, during a global crisis.

**Contribution:**

The impact of an online MBI for health care professionals amidst a pandemic has not been previously documented.

## Introduction

Health care professionals (HCPs) are typically under a great deal of stress because of the nature of their work and environment. The pressure they experience stems from emotionally taxing content, long working hours and minimal control over the environment (Aherne et al. 2016). Continuous exposure to these stressors may lead to burnout (Chirico, Nucera & Magnavita 2021). Burnout is a work-related syndrome that consists of chronic emotional exhaustion (EE), cynicism and a lack of personal accomplishment (PA) (West et al. 2016). Burnout negatively influences medical professionals’ efficiency and therefore their overall health care (Liebenberg et al. 2018).

Globally, practising medicine has become more challenging (Hollnagel, Braithwaite & Wears 2019). However, the current medical crisis brought on by coronavirus disease 2019 (COVID-19) is an unexpected global challenge at many levels (Lambert et al. 2020). At the time of the study, more than 7000 HCPs had died globally from the virus and South Africa’s infection rates amongst HCPs were rising rapidly. Further, Lambert et al. found that the measures to contain the pandemic have in themselves intensified socio-economic and mental health problems. In this context, frontline health care workers also became vulnerable to the emotional impact of COVID-19 (Serafini et al. 2020).

Spiritual practices such as meditation that induce inner growth through reflection have been found to be effective in reducing stress and burnout, and in increasing health benefits, especially during the COVID-19 pandemic (Chirico & Nucera 2020). Formal meditation practices are known to be one of the ways to enhance a sense of mindfulness (Behan 2020). Mindfulness is a term used to describe practices and characteristics of attention, awareness and acceptance (Van Dam et al. 2018). The conceptual framework underlying these qualities is a process called positive reappraisal coping. This is a cognitive strategy for reframing a situation to see it in an objective light (Folkman & Moskowitz 2000) by making one aware that thoughts are transient, psychological events rather than reflections of absolute reality. Mindfulness thus allows for a unique perspective to viewing stressful situations and the meaning attached to them.

However, imbuing mindfulness through standard 8-week programmes like the Mindfulness-Based Stress Reduction (MBSR) programme can be challenging because of time constraints (Demarzo et al. 2017). In a systematic literature review of brief mindfulness practices for HCPs, Gilmartin et al. (2017) found that shorter mindfulness-based intervention (MBI) programmes (≤ 4 h) also reported positive changes in stress levels, resiliency and burnout symptoms. These findings are encouraging, as HCPs in resource-constrained environments need a feasible and accessible MBI, considering their added constraints.

Although there is emerging evidence indicating the effectiveness of brief online MBIs for health professionals (Ma et al. 2018), more robust mixed methods research designs are needed (Moore et al. 2020). To address this gap in the literature, this study adopted a mixed methods study design, including a phenomenological perspective. Furthermore, the effects and experiences of the process of implementing a brief online MBI for HCPs in diverse, heterogeneous, multicultural settings and amidst a global pandemic have not been previously documented. Hence, this study aims to investigate the impact of a brief online MBI on the experience of stress, burnout and mindful awareness of diverse, multicultural, inter-vocational health professionals training and working during the COVID-19 crisis.

## Methods

### Setting

The context of the study is the overburdened, under-resourced health care system in South Africa during a global pandemic. During the study, the government implemented lockdown measures to restrict the movement of people, as the infection rates peaked with an average of 8000–10 000 new infections a day. However, even before the pandemic, the existing situation in the country had been labelled a health crisis (Breakfast 2018) because of the grim state of public health facilities.

### Study design

A phenomenological, participatory evaluation research intervention study was conducted with a mixed methods design using both qualitative and quantitative methods equally. The quantitative design had a pre-assessment and post-assessment component, which allowed paired comparison. The qualitative design comprised open-ended questions related to stressors, challenges and experiences before and after the online MBI. The qualitative data was analysed using interpretative phenomenological analysis (IPA). Ongoing enquiries were conducted for each component of the online MBI using both the online and WhatsApp Messenger platforms.

### Sampling strategy and study population

The invitation to participate in the study was posted on the intern, medical student, registrar and health care professions social media platforms such as WhatsApp groups and Facebook pages. Interested colleagues were encouraged to share the invitation with fellow colleagues. Thus, purposive snowball sampling was used. Snowball sampling has been found to work well when looking for information-rich sources of data from a sample with specific characteristics (Naderifar, Goli & Ghaljaei 2017). Informed consent was obtained from all participants prior to participation in the study.

The sample size was computed and verified using the software GPower 3.1, which noted that a sample size of 34 would be adequate to answer the research question, taking into consideration the research design and depth of study, with an effect size of 0.4 and a power of 0.8. Initially, 65 participants met the criteria and signed up for the study whilst 55 attended all four sessions, but only 47 completed all the required assessments. The criteria included being over the age of 18, a practising health professional, student or intern, and working within South Africa at the time of the study.

### Mindfulness intervention

A 4-week mindfulness-based programme compiled by a clinical psychologist and mindfulness teacher was adapted with permission for a brief four-session online MBI (Watkin n.d.). The programme was facilitated by two of the authors (IO & VSS) and conducted via the collaborative cloud-based video conferencing platform Zoom by two qualified and experienced mindfulness teachers. It consisted of four 1-h group sessions, as outlined in Table 1.

**TABLE 1:** Outline of the online mindfulness-based intervention.

Week	Online mindfulness-based intervention
**Week 1**	Raisin exercise and inquiry
	Body scan and inquiry
	Input on autopilot, mindfulness, poem and discussion
	3-min breathing space and inquiry
**Week 2**	3-min breathing space
	Awareness of breath and inquiry
	Yoga and inquiry
	Input on breath and body awareness, poem and discussion
	Walking meditation and inquiry
**Week 3**	3-min breathing space
	Review of home practice
	Awareness of thoughts and inquiry
	Input on stress, acceptance, allowing and letting be, poem and discussion
	3-min breathing space, action and inquiry
**Week 4**	3-min breathing space
	Body scan and inquiry
	Input on empathy, compassion and self-care, poem and discussion
	Loving kindness meditation

*Source:* Watkin, M., n.d., *Mindfulness-based cognitive therapy 4*, unpublished manual.

### Data collection

All participants completed the following validated assessments before and after the online MBI: the Mindful Attention Awareness Scale (MAAS) (Kotzé & Nel 2016), the Perceived Stress Scale (PSS) (Makhubela 2020) and the abbreviated Maslach Burnout Inventory (aMBI) (Riley, Mohr & Waddimba 2018). The MAAS has 15 items that measure trait mindfulness characteristics such as awareness of what is taking place in the present as opposed to being on autopilot (Brown & Ryan 2003). The PSS is a 10-item measure that ascertains the degree to which events in the last month in one’s life are reported as stressful. The aMBI is a nine-item self-report scale widely used to assess burnout. It has three subscales, namely emotional exhaustion (EE), depersonalisation (D) and personal accomplishment (PA). Higher scores for EE and D and a lower score for PA indicate a higher level of burnout.

Before, during and after each of the four weekly sessions, questions that explored the process and impact of the intervention on participants’ personal and professional life, including stress management, emotional regulation and how they responded to patients and work stressors, were posed via the Chat box on the Zoom platform. To counter the possibility of a positive bias towards mindfulness, questions on its challenges, drawbacks and limitations were also included. This part of the study was based on a hermeneutic-phenomenological, participatory evaluation framework. This means that every aspect of the intervention included input from the participants, and that the process was transparent and encouraged reflexivity of all the different sessions in the formal programme.

### Data analysis: Qualitative data

The qualitative data was analysed using interpretative phenomological analysis (IPA). This entails examining how participants process their experiences by looking for recurring themes (Smith & Osborn 2008). It involves the double hermeneutic of participants endeavouring to make sense of their experiences and the researcher seeking to understand the ways in which they did so.

The transcripts from the online questionnaires sent via theonline service SurveyMonkey (http://www.surveymonkey.com) and the online focus group discussions from the recorded sessions on Zoom were analysed by the principal investigator using themes that arose from the data. IPA was conducted by first doing an in-depth reading of each case and attempting to understand the individual’s perspective as much as possible by reading the pre-assessment questionnaires and comments in response to specific questions asked during the Zoom sessions. The transcripts were then uploaded to NVivo 12. NVivo 12 is a qualitative data analysis application used to create nodes and themes, and check for frequency of words to help guide the process before moving on to look for patterns of convergence and divergence. This process was followed by two authors (IO & SH) independently and then common themes thereafter discussed, collated and verified with the third author (VSS). Once consensus was reached, these themes were shared with the participants in the form of individualised written feedback as well as in a group setting during a feedback Zoom meeting because of the participatory nature of the study.

### Data analysis: Quantitative data

The research data obtained from the assessment tools (MAAS, PSS and aMBI) were primarily analysed within a quantitative framework using univariate and multivariate analyses. Data was entered from Microsoft Excel into Stata 15.1. A *p*-value < 0.05 was considered statistically significant with a 95% confidence interval. Descriptive statistical analysis of the data (means, standard deviations, ranges, frequencies and scores) was initially conducted prior to conducting inferential statistics. The paired samples *t*-test was used to assess the impact of the online MBI. Pearson’s chi-squared test and Fisher’s exact test of association were used.

### Ethical considerations

Ethical approval was obtained from the Humanities and Social Science Research Ethics Committee (HSSREC/00000848/2019) of a large South African university. Informed consent was obtained from all participants prior to participation in the study.

## Results

The sample consisted of 46% medical doctors and trainees, 16% psychologists, 14% physiotherapists, 14% occupational therapists and 10% other allied health care workers, which included a podiatrist, chiropractor and two radiographers. In terms of medical trainees, there were five medical interns and one final year medical student. Health care professionals from both private practice and government hospitals (urban and rural) participated in the study. The participants were from KwaZulu-Natal (68%), Gauteng (21%), Western Cape (9%) and Limpopo (2%). The median age was 34 with an interquartile range (IQR) of 18 years. The least experience was 6 months (medical student), and most was 33 years (health professional). No significant correlations were found between participants of different genders, ages, cultures or years of experience with regards to their levels of stress, burnout or trait mindfulness.

The Cronbach’s alpha of all the instruments was found to be comparable to previous validation studies. The Cronbach’s alpha for the MAAS was 0.9 compared to 0.87 (Brown & Ryan 2003), and the same was found for the PSS at 0.8 (Maroufizadeh et al. 2018). The Cronbach’s alpha for each subscale of the aMBI was calculated, but overall burnout was taken as the sum of EE and D, which was found to be around 0.81 in previous studies (Shaikh et al. 2019), whist in this study was found to be 0.79. Table 2 illustrates the comparison of the pre- and post-assessment mean, standard deviation, effect size, direction of the effect size and the standard deviation of the effect size.

**TABLE 2:** Comparison of pre- and post-assessment results.

Variable	*n*									Effect size				*p*
		Pre				Post				(post-pre)				
		mean	s.d.	median	IQR	mean	s.d.	median	IQR	mean	s.d.	median	IQR	
MAAS	47	3.5	0.83	-	-	3.94	0.75	-	-	0.40	0.76	-	-	< 0.001
PSS	47	21.1	6.83	-	-	15.26	5.38	-	-	−5.89	7.30	-	-	< 0.001
EE	47	10.3	4.86	-	-	8.89	4.6	-	-	−1.38	4.6	-	-	0.04
D	47	-	-	2	0–4	-	-	1	0–3	-	-	0	−2–0	0.15
PA	47	-	-	15	13–16	-	-	16	14–17	-	-	1	0–3	0.002

s.d., standard deviation; MAAS, Mindful Attention and Awareness Scale; PSS, Perceived Stress Scale; EE, emotional exhaustion; IQR, interquartile range; D, depersonalisation; PA, personal accomplishment.

Pre- and post-design quantitative research on medical trainees and HCPs who participated in the 4week online MBI displayed positive effects on the self-report measures completed, such as the MAAS, which is usually indicative of being more present and not being on an autopilot state. The mean increased from 3.5 to 3.9 with a *p*-value of < 0.001, as seen in Table 2. There was a significant decrease in the PSS and subsequent negative emotions related to perceived stress after the online MBI. Both the before and after means of the PSS fell in the moderate range, but the change in scores were significant, dropping from a mean of 21.1–15.26 with a *p*-value < 0.001. The MBI, as mentioned, has three subscales to measure burnout, namely EE, D and PA. A decrease in EE and D would be indicative of reduced burnout, as is seen in the results. Emotional exhaustion reduced from a mean of 10.3–8.89, which was a significant finding, with a *p*-value of 0.04. The means of D and PA could not be calculated because of the skewed distributions and presence of outliers; thus, the IQR and median were used instead. Depersonalisation reduced from a median of 2 to 1 after the online MBI; however, these findings were not significant. The PA scores did increase significantly from a median of 15 to 16 with a *p*-value of < 0.002, alluding to the participants’ feelings of increased competence and the ability to make a meaningful difference through their work.

**FIGURE 1:**
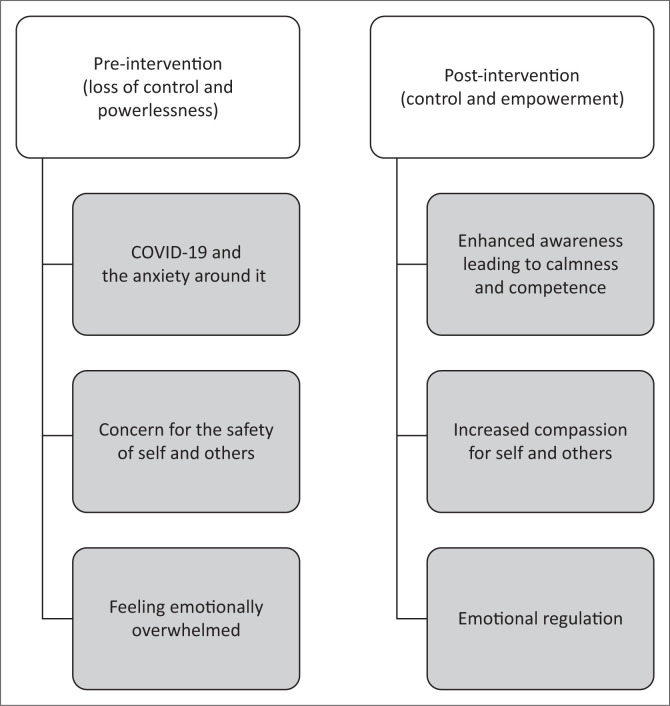
Interpretative phenomenological analysis themes and subthemes.

The qualitative results will be divided into two parts: a pre-intervention analysis with central themes of loss of control and a sense of powerlessness predominantly because of COVID-19, and a post-intervention analysis comprising themes of a sense of acquired control and empowerment because of increased awareness and mindfulness, as illustrated in Figure 1. These main themes will be explored and discussed within various subthemes. Trustworthiness was ensured by using the words of the participants to elaborate the themes and each interpretation was checked with them to ensure bracketing of the researchers’ own possible bias.

## Pre-intervention analysis

### Theme 1: Loss of control and powerlessness

It was unsurprising that COVID-19 presented as a major stressor and appeared to have affected every single participant, mostly in a deleterious manner. For the theme of loss of control and powerlessness, the subthemes are the following: COVID-19 and the anxiety around it, concern for the safety of self and others, and a sense of being emotionally overwhelmed.

#### Subtheme 1.1: COVID-19 and the anxiety around it

COVID-19 was in essence seen as a crippling and harmful life-altering event, which was exacerbated by the fact that one’s mortality was highlighted, resulting in increased anxiety. These feelings were further fortified by other elements, such as a lack of resources and an overwhelming sense of powerlessness and loss of control. To further compound the psychological impact and dread caused by COVID-19, the subsequent lockdowns to prevent the spread of the disease had caused a severe economic crisis. Moreover, the limited understanding of the pandemic and how it is spread further added a sense of frustration, uncertainty and dread (Serafini et al. 2020).

In the transcripts, the word ‘COVID’ was mentioned 55 times, ‘anxious’ around 100 times and ‘stressful’ 45 times. These words became the basis of the first theme, for every participant, without exception, spoke of the impact of COVID-19 on their work and how it was hard to adjust to the new challenges that this situation presented. The participants expressed their anxieties in the following statements, in which they appear to perceive COVID-19 as a constant threat:

‘My world had been spinning out of control since the beginning of COVID and the extreme stress I was under.’ (Participant A, 42 year old, female, medical doctor)

‘Being emotionally spun out in the COVID environment at work especially … burnout and loss of enthusiasm for my work, feeling of fear and even dread when I think of work.’ (Participant B, 46 year old, female, chiroprator)

#### Subtheme 1.2: Concern for the safety of self and others

One of the main reasons cited for the elevated anxiety levels was the fear of increased health risks to self and family members, which are conveyed by the following quotes:

‘Fear that I will contract COVID again.’ (Participant C, 28 year old, female, medical intern)

‘The main stressor currently is the COVID pandemic and the fear that I will bring it home to my family.’ (Participant D, 34 year old, female, psychiatry registrar)

The participants attempted to reduce this fear of getting sick and, worse, passing on the virus to family members by isolating themselves from family during this time, which had its own set of repercussions.

#### Subtheme 1.3: Feeling emotionally overwhelmed

The participants felt emotionally overwhelmed because of limited resources, an invalidating work environment and exposure to an increasing number of deaths. According to the participants, a significant contributing factor that increased their anxiety was the fact that they were expected to work with COVID-19 patients with limited personal protective equipment and little or no support from their work environment. This increased their fear of contracting the disease, which was further heightened by witnessing high death tolls, leading to them feeling extremely emotionally overwhelmed, as evidenced in these participant statements:

‘I’m stressed about seeing patients and passing on any diseases to patients because we aren’t being provided with [personal protective equipment].’ (Participant E, 23 year old, female, community service occupational therapist)

‘Demanding patients, feeling sympathy for them and family but unable to fully help them due to lack of resources.’ (Participant C, 28 year old, female, medical intern)

The participants reported that a contributing factor to their experienced stress and heightened anxiety was working within a perceived invalidating and unsupportive work environment, as mentioned in the following statement:

‘Lack of sleep, lack of appreciation at work, feeling like a slave at work being disrespected… Given no warmth and consideration. Being unheard. Feeling out of control; that I should’ve done better, exhausted.’ (Participant C, 28 year old, female, medical intern)

Although the HCPs may be used to death and dying, it being part and parcel of their profession, the scale of deaths caused by the COVID-19 pandemic was unprecedented. This resulted in heightened guilt, helplessness and dissociating as a way of coping:

‘I see people die daily … the only way to deal with all these deaths is to be nonchalant about them.’ (Participant F, 24 year old, female, physiotherapist)

‘Guilt about death and bereavement, becoming numb.’ (Participant C, 28 year old, female, medical intern)

The overall feelings prior to the intervention appeared to be fear, helplessness and feeling emotionally overwhelmed because of the uncertainty and conditions that the HCPs were expected to work in, which affected their overall sense of competence and well-being. A way in which many of the participants chose to cope during this time was to go on autopilot and simply dissociate from the emotions they were feeling.

### Post-intervention analysis

#### Theme 2: Control and empowerment

The main themes to emerge from the post-intervention data was an increased sense of control and a feeling of empowerment, which were related to the increased ability to be mindful. An enhanced sense of awareness and present-moment focus led to an overall sense of calmness and competence, increased compassion towards oneself and others, and better emotional regulation.

**Subtheme 2.1: Enhanced awareness leading to calmness and competence:** As illustrated in Table 2, the MAAS levels increased significantly after the intervention, indicating that the participants were able to be more present and aware, and alluding to an increased ability to be mindful compared to before the intervention. These results were further fortified by the qualitative data. The word ‘awareness’ was the most-mentioned word and appeared 261 times in the transcripts, followed by the word ‘present’, which appeared 102 times. Being more aware of one’s self, thoughts and emotions appeared to lead to increased compassion towards oneself and others, being able to respond in a calmer way, regulate one’s emotions better, and feel more competent in one’s work. This is highlighted in the following statement, which depicts how self-awareness influences self-control:

‘I’m noticing that, if I become aware of the thoughts in my mind, I can be more in control in terms of directing how I respond.’ (Participant G, 30 year old, female, psychologist)

Many participants also spoke of relief at being present in the moment as opposed to looking at their actions in the past, which often led to regret and harsh self-criticism, or worrying about the future, which they claimed caused anxiety. This new way of relating to thoughts led to a feeling of calmness. The participants said the following:

‘I feel like being present in the moment allows me to have a better experience of what I am doing. I am enjoying my activities.’ (Participant H, 36 year old, female, physiotherapist)

‘I reflected on how irritable I had been prior to the intervention and just how fatigued I felt. It also brought greater awareness to my need to just be present. I’m constantly worrying about something or planning the future that I neglect the present and it’s so exhausting.’ (Participant I, 32 year old, female, psychologist)

When comparing the assessment results prior to the intervention and then immediately after it, the PSS levels dropped significantly after the attendance of the online MBI (see Table 2). These results are further understood by looking at the following participant statement:

‘I definitely kept thinking about my thoughts… How I tend to make it worse by playing it out in my mind. Now I’m trying to let go more.’ (Participant H, 36 year old, female physiotherapist)

It is understandable that health professionals are under much pressure, and feel the need to multitask and rush through their work. However, being mindful does not appear to take more time as one would fear. By being more present and engaging with increased awareness and ability to be calm, it appears that the participants saved time through increased efficiency and performance:

‘I have noticed that I was able to be more ordered in my patient treatment process. I didn’t miss any steps or need to redo anything.’ (Particpant J, 29 year old, female, podiatrist)

‘I think that a lot of the principles of mindfulness really enhance and encourage that process, not just on a personal level that you are less stressed and more peaceful, but also you become more effective.’ (Participant K, 32 year old, female, opthamologist)

This feeling of increased efficiency was also noted in the PA scale in the aMBI, as seen in Table 2. The questions that check for feelings of PA centred around feelings of being efficient at work, being able to make a difference and finding meaning in the work that they do.

**Subtheme 2.2: Increased compassion for self and others:** An increased awareness changed the way in which the participants related to their thoughts and feelings, and led to an enhanced sense of meta-awareness, that is, being aware of oneself and identifying one’s own needs. This appeared to lead to taking a kinder approach to oneself and others. The increased self-compassion resulted in more self-care behaviours being adopted, which is critical as these behaviours sustain the decrease in stress and maintain the lessons learnt from the mindfulness practice. Self-compassion and self-care were evident in the following statements:

‘Being present without judgement has really stayed with me these last couple of weeks as I am so hard on myself at times – mindfulness has helped with this.’ (Participant L, 26 year old, female, occupational therapist)

‘It has taught me how to not want to constantly please people around me at my own expense. I want to do the best for my patients, so I give 100%, but I have to acknowledge that I fill my own cup first.’ (Participant H, 36 year old, female physiotherapist)

An increase in compassion towards patients and peers was observed in the following statements:

‘Instead of being angry with patients because the systems are not working, I started to engage with them on a more personal level and realise the challenges they are going through. This has humbled me.’ (Participant M, 56 year old, female, radiographer)

‘I have been able to respond with more care and kindness to my colleagues who have been needing assistance, despite my own workload.’ (Participant N, 39 year old, female, educational psychologist)

‘Accepting and recognising that others are going through an emotionally harrowing time, and being able to show patience and compassion.’ (Participant B, 46 year old, female, chiropractor)

**Subtheme 2.3: Emotional regulation:** The awareness of emotions leads to enhanced emotional regulation skills, as seen in the EE subscale scores of the aMBI (see Table 2), which showed a significant reduction in EE. These results were explained by a participant as follows:

‘I was more aware of reacting emotionally to something that triggered a feeling of anger, was able to just ground myself and eventually managed to react with calmness.’ (Participant O, 40 year old, female, neonatologist)

## Discussion

The focus of this study was to investigate the impact of a brief online MBI on stress, burnout and mindful awareness of HCPs working and training during the COVID-19 crisis. Pandemics are a significant public health threat that demand changes in most aspects of everyday life (Bowman 2020). Frontline health care workers in fact bore the brunt of the pandemic, as they became the ‘defence force’ against this new enemy. The general sense from management and government appeared to be that HCPs knew what they signed up for and that they must just do what needs to be done with whatever resources they have. The rising patient admissions and increasing workload exacerbated HCP’s anxiety. Compounded by a lack of support from management and no strategies in place to prioritise the well-being of the overworked HCPs.

By taking into consideration the high stress levels, the poor coping mechanisms and the under-resourced conditions in which the HCPs worked, one might argue that the environment in which they are expected to function should be changed. Whilst that may not occur as quickly as we would like it to, a more constructive approach of altering the HCPs’ perspective of the situation by reframing their mindset was adopted. This new perspective had a subsequent impact on their emotions, behaviour and resilience, as observed in this study and previous research (Pillay 2020). In essence, this study positively enhanced the HCPs’ perceptions of and responses to highly stressful contexts.

The quantitative results confirmed that our sample consisted of predominantly highly stressed individuals who were vulnerable to burnout with low levels of mindful awareness and present-moment focus. Uncertainty is known to raise anxiety, even in psychologically healthy individuals (Shigemura et al. 2020). Medical professionals develop a manner of coping with uncertainty and death, as they are regarded as unwelcome parts of the profession. However, with regards to COVID-19, the number of deaths significantly increased as the world was in the process of learning how to respond to this disease, and everything changed in a very short period of time. This resulted in heightened guilt and helplessness, as illustrated by the findings of this study.

The study found notably significant post-intervention changes. These were reduced stress levels, lower levels of EE, a feeling of being more present and aware, and overall enhanced feelings of self-competence, as evident in the data presented (Table 2). This benefitted the HCPs by helping them to be more aware of their own thoughts and feelings, resulting in being more emotionally regulated, responding more mindfully to stressors, and being more compassionate and caring to self and others (Scheepers et al. 2019). Whilst the challenges of dealing with the unknown and a lack of control of the future were still present, what the practitioners learnt was that they could work and function within the present, which reduced anxiety over an unpredictable future. In practice, this meant that as opposed to being focused on an assumed apocalypse, the HCPs changed their perspectives to the here and now and what they could control. This change was found to be liberating, as it removed the anxiety that the feelings of lack of control and helplessness triggered, and reinforced their ability to revert to their pre-COVID medical competence and professional confidence. The encouraging factor was that this was achieved through a brief 4-week intervention as opposed to longer 8-week programmes (Demarzo et al. 2017), and via an online version instead of the normal face-to-face sessions (Moore & Hutchinson 2020). Although there is some research to show the benefits of brief online MBIs (Ma et al. 2018), this is the first major descriptive study known to the authors to show the process and impact of an online MBI during such intensely stressful times with a group of heterogenous, interdisciplinary HCPs.

The significant decrease in stress levels, with a concomitant increase in the sense of empowerment and a heightened sense of perceived control, was further validated by the qualitative findings. Not only did the HCPs speak of enhanced well-being, but also of increased efficiency and resilience that were noted in both their objective and subjective sense of competence. Compassion was another pivotal theme that changed the way the HCPs related to themselves and others. Previous research has shown that the medical community is now increasingly acknowledging that competent professionals are committed to taking care of their own mental health needs as well as those of their patients (Knudsen et al. 2020). The prioritising of one’s own well-being not only reduces stress, but also increases performance and enhances one’s ability to relate to others (Knudsen et al. 2020).

This study’s findings not only align with previous findings, but also contribute to the growing body of evidence-based studies that confirm the effectiveness of mindfulness as a therapeutic intervention and in facilitating practitioner growth and skills development (Daya & Hearn 2018). Thus, we emphasise the importance of practitioner self-awareness of his or her interactions with patients, which reinforces the adoption of a more patient-centred approach. Therefore, the adoption of mindfulness has multifactorial benefits.

Little research to date has focused specifically on how HCPs experience mindfulness within multicultural, interprofessional settings and during situations of intense stress and crisis (Knudsen et al. 2020). This study had a cross-sectional sample with a range of different health professionals of differing years of experience and training working under varied circumstances during the COVID-19 crisis. Culture, language and power or hierarchy were also considered, but the results were found to be universal and we were able to transcend these differences. Perhaps the online Zoom platform facilitated a focus on the MBI practices and not on each other’s differences and diversity. These findings may also be attributed to the collective group experience of a crisis whilst practising weekly mindfulness sessions together.

## Strengths, limitations and future directions

This study has some major strengths, including the innovative method of online delivery using Zoom and finding significant results, even with a brief time period of just 4 h. The mixed methods design provided a more comprehensive understanding of the underlying factors contributing to the changes in the participants’ stress and burnout scores. As with all qualitative research though, generalisability can be a challenge (Atieno 2009). This was taken into consideration by adding the quantitative component. By using IPA, we were able to explore the participants’ experiences pertaining to the process of imbuing mindfulness and obtain rich descriptions of these experiences.

The number of participants was not large enough and thus lacked the power to find significant correlations between factors such as the ability to be mindful in relation to age, years of experience, vocation and so on. Thus, separate studies with larger numbers investigating these are recommended. However, one can also infer from this study the possibility that mindfulness transcends these factors and is applicable to everyone, thereby strengthening the argument that it should be considered for inclusion in the medical training programme.

Dropout rates, also known as experimental attrition, are a common challenge in intervention studies, with numbers that have been known to fall at 15% – 20% (Cramer et al. 2016). We had a dropout rate of 28%. This was possibly because of the erratic work schedule, long hours and stressful time that the HCPs were experiencing during the COVID-19 pandemic. We did offer two classes a week of individual make-up sessions, but were still unable to accommodate everyone’s schedules. Organisational support and incentives to attend would be highly beneficial to improve attendance and subsequently self-care in HCPs.

## Conclusions and recommendations

The implications of the study point towards the feasibility and effectiveness of using MBIs as part of employee wellness programmes (Chirico & Magnavita 2019). This study recommends that MBIs be incorporated into HCP training to create opportunities for students to imbue mindfulness into their clinical training, thereby encouraging a positive focus on mental health and well-being, and more especially as training for possible future medical crises.

As the pressures placed upon our HCPs escalate, the focus on online MBIs is timely and can be seen as lifesaving. Research advocates mindfulness training as a practical tool for the promotion of self-care, well-being and increased competency (Irving, Dobkin & Park 2009). This study skilfully elucidates how one can apply a system of psychological management to an under-resourced and overburdened public health system with proven effectiveness and with minimal resources and cost, therefore making it more likely to be adopted by health care systems. In essence, the outcome should be professionals who are more resilient and emotionally higher functioning, and this may result in improved service delivery as well as improved retention rates within the sector. This study critically demonstrates the feasibility of implementing a brief online MBI and its significant impact on burnout and stress in a worldwide crisis situation.
